# Gene Therapy in Rare Respiratory Diseases: What Have We Learned So Far?

**DOI:** 10.3390/jcm9082577

**Published:** 2020-08-08

**Authors:** Lucía Bañuls, Daniel Pellicer, Silvia Castillo, María Mercedes Navarro-García, María Magallón, Cruz González, Francisco Dasí

**Affiliations:** 1 Research group on Rare Respiratory Diseases (ERR), Department of Physiology, School of Medicine, University of Valencia, Avda. Blasco Ibáñez, 15, 46010 Valencia, Spain; lucia.banyuls.soto@gmail.com (L.B.); dpellicerroig@gmail.com (D.P.); mariamagallon94@gmail.com (M.M.); 2 Research group on Rare Respiratory Diseases (ERR), Instituto de Investigación Sanitaria INCLIVA, Fundación Investigación Hospital Clínico Valencia, Avda. Menéndez y Pelayo, 4, 46010 Valencia, Spain; sccorullon@gmail.com (S.C.); mer_navarro2002@yahoo.es (M.M.N.-G.); cruz.gonzalez@uv.es (C.G.); 3 Paediatrics Unit, Hospital Clínico Universitario de Valencia, Avda. Blasco Ibáñez, 17, 46010 Valencia, Spain; 4 Pneumology Unit, Hospital Clínico Universitario de Valencia, Avda. Blasco Ibáñez, 17, 46010 Valencia, Spain

**Keywords:** gene therapy, rare respiratory diseases, alpha-1-antitrypsin deficit, cystic fibrosis, primary ciliary dyskinesia

## Abstract

Gene therapy is an alternative therapy in many respiratory diseases with genetic origin and currently without curative treatment. After five decades of progress, many different vectors and gene editing tools for genetic engineering are now available. However, we are still a long way from achieving a safe and efficient approach to gene therapy application in clinical practice. Here, we review three of the most common rare respiratory conditions—cystic fibrosis (CF), alpha-1 antitrypsin deficiency (AATD), and primary ciliary dyskinesia (PCD)—alongside attempts to develop genetic treatment for these diseases. Since the 1990s, gene augmentation therapy has been applied in multiple clinical trials targeting CF and AATD, especially using adeno-associated viral vectors, resulting in a good safety profile but with low efficacy in protein expression. Other strategies, such as non-viral vectors and more recently gene editing tools, have also been used to address these diseases in pre-clinical studies. The first gene therapy approach in PCD was in 2009 when a lentiviral transduction was performed to restore gene expression in vitro; since then, transcription activator-like effector nucleases (TALEN) technology has also been applied in primary cell culture. Gene therapy is an encouraging alternative treatment for these respiratory diseases; however, more research is needed to ensure treatment safety and efficacy.

## 1. Introduction

### 1.1. Why Gene Therapy?

Since the discovery of the way DNA transmits information from parents to progeny, medical research has focused on finding a way to reverse malfunctions to improve the quality of life in people suffering genetic-related conditions. Advances in the understanding of gene interactions and their physiological consequences have put gene therapy in the spotlight as the key for creating personalized medicine.

Gene therapy can be described as a disease treatment that introduces exogenous genetic material such as plasmid DNA, antisense oligonucleotides, mRNA or peptide nucleic acids into cells or tissues with the aim of treating hereditary or acquired genetic disorders. In essence, it is the use of nucleic acids as a drug to obtain therapeutic benefits for the patient [1].

### 1.2. A Brief History of Gene Therapy

When Theodore Friedmann introduced the idea that gene therapy could treat monogenic diseases in 1972 [2], the novel gene therapists started facing the barriers that the body has to protect us from foreign DNA. We are currently on the race to treatments that promise a one-dose administration and a stable expression of a protein of interest. Thus, through the last 50 years, gene therapy development has been facing countless challenges and feeding back on itself until it has reached the first glimpses of clinical uses. At the beginning of the 1990s, clinical assays using viral vectors started with high optimism, but a series of catastrophic events ended up with the death of the first volunteer during gene therapy clinical trials, Jesse Gelsinger [3]. That particular event reached far beyond the scientific community and severely damaged the already controversial studies on human research. Lack of support from the institutions during the beginning of the millennia revived the debate on the concerns of gene editing. Thus, in order to improve the safety of the possible treatments, basic science continued studying the mechanisms of the viral vectors used and developed synthetic particles that could mimic the process of delivering genetic material without the risk of letting a virus get out of control. The deeper knowledge on the metabolic routes allowed more and more clinical trials, and after exhaustive tests, the FDA approved the first gene therapy in 2017, Luxturna from Novartis [4]. To date, 17 cellular and gene therapy drugs have been FDA-approved and listed in the Office of Tissues and Advanced Therapies (OTAT), and there are hundreds of clinical trials on the way due to the broad range of available viral and non-viral vectors and techniques to modify genetic information. At the same time, we have gained a greater understanding of molecular bases and genetic causes of many diseases, which encourages gene therapy applications targeting key cells and organs for almost any genetic disease.

One of the most promising branches of gene therapy uses avant garde gene-editing techniques such as CRISPR Cas9. The appearance of these techniques in scientific papers saw exponential growth from 2012 on, and clinical trials have started to appear during the recent years. In 2018, the first human in vivo test using CRISPR technology was performed by Editas with the drug EDIT-101 [5], which used associated adenoviruses to introduce the genetic material for editing a mutation causing Leber congenital amaurosis 10. Results from the clinical trial were satisfactory, and the first patient has received treatment in 2020 during the clinical trial phase II [6]. The events are promising; nevertheless, especially concerning are the possible off-target effects, mutations that appear in erratic locations, and the transmission of those mutations to the germ cells, which could end up in the progeny and cause several problems. History has shown that further tests are needed to prove the safety of these therapies in the long term.

### 1.3. Gene Therapy Challenges in Rare Respiratory Diseases

Many respiratory conditions have a genetic origin that can be addressed by gene therapy techniques. Lungs are an attractive organ for gene therapy as vector delivery can be performed directly into the lungs via the air with nebulization, bronchoscopy, pleural administration, or through the blood via intravenous administration [7,8,9]. Nevertheless, the complexity of the lung structure places certain physical and chemical barriers to vector delivery, especially for viral vectors. The airway epithelium is covered by a mucus layer whose function is to trap and clear exogenous material, including gene therapy vectors. Moreover, most of the vector’s cell receptors are located in the basolateral surface of the cells. Epithelial cells form tight junctions between them, making it very difficult for vectors aiming at the basolateral side to reach their destination [10,11]. Given the exposure of airways to the external environment, lungs have natural protection from the immune system against viruses and other exogenous agents, which increases the possibility of adverse effects due to inflammatory response [12]. In addition, in many respiratory diseases, these barriers are often exacerbated: mucus production is increased, and there is a proinflammatory basal state. The constant renewal of epithelial cells is also challenging, provoking the need for repeated administration, which increases the possibility of activating the immune system [13]. One possible solution could be to target stem cells with integrating vectors, but there are some associated concerns such as integrational mutagenesis and the low efficiency of stem cell transduction in vivo due to their location in the basal surface of the tissue. Preclinical studies are also challenging, due to the difficulty of finding an animal model that mimics human lung cell biology and function, with the result that therapies successful in animals are complicated to translate to humans [14]. Despite all these obstacles, there have been many attempts to develop both in vitro and in vivo gene therapy, using all kinds of vectors and strategies to treat different respiratory diseases [14].

In this review, we aim to provide a general overview of the state of the art of gene therapy applied to three of the most common rare respiratory diseases such as cystic fibrosis (CF), alpha-1 antitrypsin deficiency (AATD), and primary ciliary dyskinesia (PCD). We summarize the vectors used and developed to treat these diseases, the administration routes implemented, and the outcomes obtained, successful or otherwise. Among the approaches used to address these respiratory conditions are augmentation therapy introducing a wild-type copy of the gene, silencing defective gene expression, directly removing the mutation, and trying to edit and correct the nucleotide sequence. Finally, we shed some light on future perspectives for gene therapy and lung disease.

## 2. Gene Therapy Strategies

### 2.1. Transfection Vectors

The effective delivery of genetic material inside cells of interest can be achieved by a myriad of methods. Focusing on gene editing, the vectors have to be capable of inserting Cas9, the associated tracrRNA (trans-activated Clustered Regularly Interspaced Short Palindromic Repeats (CRISPR) RNA) and gRNA (guide RNA). Some approaches are more suitable for in vitro and others for in vivo therapies. However, the key aspect for selecting between different methods is undoubtedly the kind of project carried out [15].

### 2.2. Viral Vectors

Viral methods exploit the innate abilities of viruses to insert the desired genetic material, although their generally high efficiency in delivering and expressing genetic material has a major drawback: they require at least a level II biosafety laboratory and safety procedures to ensure no major complications arise when manipulating the virus. Viral methods are generally divided into genome-integrative and non-genome integrative, and their advantages and disadvantages are shown in the table below (Table 1) [16]. The last column indicates the existence of studies using the virus for CF, AATD, or PCD. Strict regulations and safety concerns only allow few viral vectors to reach the clinical trial phase. In CF and AATD, there are several studies and clinical trials explained in their corresponding sections. In PCD, there is no information about clinical trials using viral vectors.

### 2.3. Non Viral Vectors

Non-viral methods take advantage of different chemical and physical properties to pierce the membrane and insert the genetic material or proteins. The optimal method depends on the cell or tissue to be transfected. Naked DNA may be inserted directly in the cells, using a physical method; however, its lack of stability and low permeability through the membrane due to its negative charges cause a very low expression of the gene of interest and can cause inflammatory responses [24]. For these reasons, chemical coating systems are highly recommended or necessary in some cases. This issue has been overcome using different creative solutions such as physical methods, inorganic molecules, and other biocomponents. Combining different methods best optimizes the transfection process [25].

### 2.4. Physical Methods

Physical methods (Table 2) are mainly used to penetrate the cell membrane barrier. This can be done by briefly creating holes and making it permeable for a sufficient amount of time to allow the material of interest to enter the cytoplasm [26].

Some of these methods of mammal cell modification can only be performed in cell culture and they are useful in understanding the mechanisms of the disease, while others are used to modify animal models and have the potential to be used in medical trials. Decisions about their use are made depending on the size of the plasmid, the cell type and organization, or the aim of the project.

The use of the techniques used in rare respiratory diseases is expanded in each of the sections of the diseases of the article.

There are different approaches for improving the efficiency of the physical methods; binding the DNA to different structures increases the stability and reproducibility of results. Conventionally, they are stratified into inorganic particles and biocomponents.

### 2.5. Inorganic Particles

Inorganic particles (Table 3) bind to DNA due to their chemical properties and allow better insertion control than naked DNA; they are also generally cheap to produce and easy to store [32].

### 2.6. Biocomponents

Biocomponents (Table 4) are biodegradable structures enclosing DNA or biopolymers that interact with their negative charges. Their versatility and chemical properties are highly attractive for gene therapy use. Their main benefit against inorganic particles is that their degradation products are organic and normally enter the citric acid cycle after use, lowering toxicity and increasing biosafety.

### 2.7. Gene Editing Techniques

**Figure 1:**
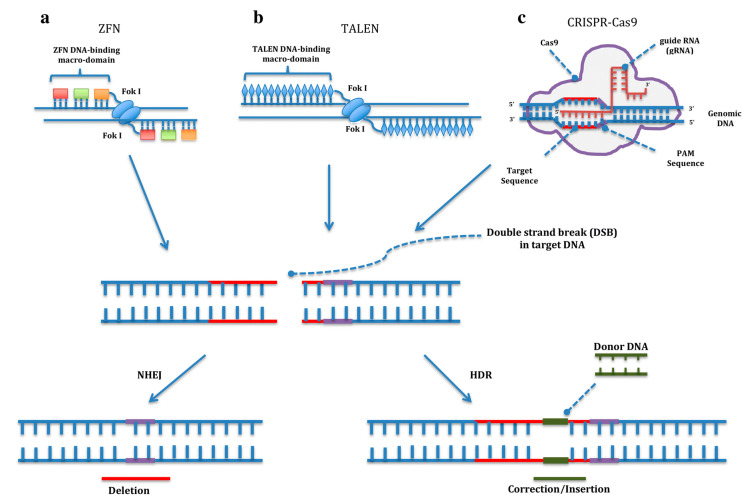
Genome editing with zinc finger nucleases (ZFN) (**a**), transcription activator-like effector nucleases (TALEN) (**b**) and CRISPR/Cas9 (Clustered Regularly Interspaced Short Palindromic Repeats). (**c**) First, a double-strand break (DSB) is produced in the target sequence, and then, it is repaired by non-homologous end joining (NHEJ) or homology-directed repair (HDR). NHEJ produces random insertions and deletions useful for knockout experiments, while HDR results in a precise modification of the target sequence. Reproduced with permission from Torres-Durán et al. [59] under the terms of the Creative Commons Attribution 4.0 International License (http://creativecommons.org/licenses/by/4.0/).

Gene editing techniques allow researchers to manipulate genes according to their interests. These tools are based on a domain that specifically binds to a DNA sequence associated with an endonuclease domain that produces a double-strand break (DSB). Then, the damage on the genome is repaired by the cell mechanisms. During the process, a small insertion or deletion can be produced or a single nucleotide can be precisely changed due to the presence of two alternative pathways to repair the DSB: non-homologous end joining (NHEJ) or homology-directed repair (HDR). The first, NHEJ, generates random insertions or deletions to repair the DSB, which results in disruption of the gene function useful for gene knockout experiments, while the HDR pathway repairs the DNA damage by copying a homologous template. By delivering a DNA donor encoding the sequence of interest into the cell, the genome can be precisely edited [43] (Figure 1).

### 2.8. Zinc Fingers and Transcription Activator-Like Effector Nucleases

The first gene editing tool to be developed was zinc finger nucleases (ZFN). Cys2His2 zinc fingers proteins are DNA-binding proteins with a particular structure that interact with a specific triplet on the DNA. By varying the amino acid sequence of the finger, different DNA triplets can be recognized. Due to the modular nature of zinc fingers, different fingers can be linked together to recognize a specific DNA sequence. In addition, zinc fingers are fused to the proteolytic domain of nuclease FokI to obtain the ZFN editing tool [44] (Figure 1).

Another approach in gene editing is transcription activator-like effector nucleases (TALEN), based on the transcription activator-like (TAL) effectors from plant pathogenic Xanthomonas spp. The bacteria use TAL effectors to bind the host DNA and activate the expression of certain genes. They consist of modules of 33–35 amino acids that recognize one specific nucleotide. Through creating a modular protein by fusing different TAL effectors, virtually any sequence on the genome can be targeted. These proteins have been fused to the endonuclease activity of FokI, just as in ZFN, to develop the TALENs gene editing tool (Figure 1). The only requirement of TALENs is that the target sequence must start with a thymine. Since two domains of FokI are needed to produce a DSB, both ZFN and TALENs must be designed in pairs, binding the two complementary DNA strands while leaving enough space for FokI to dimerize [45].

### 2.9. CRISPR/Cas9

The most recently emerged gene editing tool is the CRISPR/Cas9 system (Clustered Regularly Interspaced Short Palindromic Repeats). It is an adaptation of an adaptive immune system against viruses from bacteria and archaea. In 2012, two independent publications pointed to the CRISPR/Cas9 system as a potential gene editing tool, describing it as a two-RNA structure targeting a specific DNA sequence and the Cas9 nuclease that cuts it [46,47]. It is this simplicity that has boosted the spread of CRISPR/Cas9 among thousands of laboratories worldwide. Basically, it consists of (1) a single guide RNA (sgRNA), formed by two RNA molecules: a crRNA with approximately 20 nucleotides complementary to the target sequence, fused to an invariable trans-activating crRNA (tracRNA), and (2) the Cas9 protein that contains two nuclease domains, an endonuclease domain named HNH for having characteristic histidine and asparagine residuesand another domain named Ruv-C, producing a DSB. The main drawback of the CRISPR/Cas9 system is that a protospacer adjacent motif (PAM) with an NGG sequence is required at the end of the target site. After binding, the Cas9 protein cuts three bases upstream of the PAM sequence [48] (Figure 1).

In addition to knock-in and knockout experiments such as ZFN and TALENs, different applications have been developed by modifying the Cas9 protein function. Endogenous genes can be activated or repressed using CRISPR activation (CRISPRa) [49] or CRISPR interference (CRISPRi) [50]. By fusing a dead Cas9 with other deaminase enzymes, one nitrogenous base can be transformed into another in a specific way without producing a DBS. These tools are called CRISPR base editors [51,52]. One of the main concerns regarding the CRISPR/Cas9 system is off-target activity. In order to reduce it, the widely used Streptococcus pyogenes Cas9 nuclease (SpCas9) has been engineered to improve its specificity, such as for instance the D10A Cas9, which only cuts one DNA strand, thus necessitating two proteins binding in the same locus to produce a DSB [53]. Other examples are modified Cas9 proteins such as enhanced specificity Cas9 (eSpCas) [54] or SpCas9-HF [55]. Aiming to expand the limits of the Cas9 protein, other nucleases have been reported as efficient and specific when editing human cells. Among examples are the smaller but also efficient Staphylococcus aureus Cas9 (SaCas9), which recognizes NNG(A/G)(A/G)T PAM sequence, and its variant KKH SaCas9 [56], which recognizes the NNN(A/G)(A/G)T PAM sequence, but it also nucleases different from Cas9 proteins, such as Cpf1 nucleases: AsCpf1 (from Acidaminococcus sp. BV3L6) and LbCpf1 (from Lachnospiraceae bacterium ND2006). Cpf1 nucleases are guided by a 42-nt crRNA, without tracRNA, and recognizes the TTTN PAM sequence, which is located at the 5′ end of the target sequence [57]. AsCpf1 nuclease has also been engineered to obtain new variants associated with new PAM sequences [58].

### 2.10. Cellular and Animal Models

Animal models are key to understanding the physiopathology of a disease. Rare diseases are generally caused by one or a cluster of genes that are conserved among species, so finding the orthologue of the gene of interest in an animal allows detailed studies on malfunction and unravels different approaches on how to find a therapy. Each animal model has its pros and cons, and using one or the other depends on the nature of the study to be conducted.

Murine models are the classical example of an animal model, and they are useful for their fast reproduction, relatively easy maintenance, well-studied genomes, and highly reproducible results. Focusing on respiratory diseases, mice, guinea pigs, rats, and pigs are the most frequently used models, noting that inflammatory processes are mimicked in mice. Pigs have the most similar lungs to humans due to the presence of the connective tissue, the size, and the arrangement of the lymphoid tissues in the nasopharynx [60].

### 2.11. Animal Models in CF

The most important trait for CF animal models is CFTR gene homology, so listing the animals most commonly used as CF models in this order, pigs have a homology percentage of 93% [61], while ferrets have 92% [62], sheep have 91% [63], mice have 78% [64], rats have 75.5% [65], and zebrafish have 55% [66]. CF models should also ideally have spontaneous lung disease and/or infection by the common pathogens that appear in CF patients.

Each animal has its advantages and drawbacks, and research is focused on finding a model that reproduces the physiopathology of the disease [67].

### 2.12. Animal Models in AATD

AAT is a multi-functional molecule that requires complex organisms for understanding its different roles. Murine models used in the studies provide excellent information on how AAT is created and transported, but hepatopathies, lung emphysema, and anomalous inflammatory responses do not affect mice as much as humans [68]. First, human Protein inhibitor Z mutacion (PiZ) AAT murine models were developed in 1987 and were used to measure the expression of PiZ in different tissues, mainly liver and kidneys [69]. Liver accumulation and damage was reported similar to that in humans, but pulmonary disease was not reported due to the presence of other antiproteases [70]. Due to the complexity of the murine *Serpina1*-gene locus, that presents up to six *Serpina1* paralogs, it was not until 2018 that Borel et al. created a *Serpina1*(a–e) knockout mouse to mimic AATD lung physiopathology. This mouse could be used not only for a better understanding of the formation of panlobular emphysema in AATD, but also for the genetics pathways of the disease [71]. So far, there is no murine model with concurrent lung and liver disease [72]. In silico approaches for the creation of humanized AAT mice might help to create better models for testing gene editing techniques in living organisms. Nevertheless, some of the techniques applied in mice genetic modifications, such as hydroporation, which is further explained later on the article, are not scalable to bigger animals such as pigs because the stress exerted causes fatal organ damage. New techniques developed to overcome these issues make it a better approach to find a human therapy [73].

Besides murine models, a transgenic zebrafish model has recently been developed which expresse the Z mutant form of the human AAT(Z-hAAT) and shows liver stress and decrease in AAT circulating levels. This new animal model may help explore AATD physiological bases and treatment [74].

### 2.13. Animal Models in PCD

Murine models are adequate to study the function of the cilia in an organism because of their left-right asymmetry, lungs, mucociliary clearance, and similar genetic disposition to humans. However, many mutant alleles have proven lethal due to hydrocephalus, cardiac, or kidney defects caused in neonatal or early live stages [75]. Due to the nature of the disease, which consists of a defect in an organelle, different approaches may be taken according to the nature of the malfunction to study. Unicellular organisms are useful for understanding cilia biology, among which *Chlamydomonas reinhardtii* is the most commonly used, particularly due to its wide spectrum of mutants, which exhibit different mutations. Studies using this model have furthered understanding of the basics of the cilia structure [76]. Other unicellular organisms such as *Trypanosoma brucei* and *Paramecium tetraurelia* provide different insights into motility mechanisms [77] and genetics [78]. However, complex organisms are required to understand the pathophysiology of the disease. *Drosophila melanogaster* has two types of cilia, with 9 + 0 (in cells involved in hearing and coordination) and 9 + 2 (in sperm cells) structures, and it shares transcription factors with humans [79]. However, cilia assembly mechanisms are different, and mucociliary clearance is absent.

*Xenopus laevis* and *tropicalis* are very useful to study these traits because of their ciliated epidermis, which creates a flow from the head to the tail [80], and additionally, left–right asymmetry developmental studies can be performed due to their short developmental cycle [81]. Meanwhile, zebrafish (*Danio rerio*) is a well-known model for left–right asymmetry [82] and is suitable for gene editing techniques such as the ones previously mentioned; however, it has no lungs, and some of the cilia-related genes are duplicated, which can create confusion due to genetic redundancy.

## 3. Gene Therapy in Rare Respiratory Diseases

### 3.1. Cystic Fibrosis

Cystic fibrosis (CF) is an autosomal recessive disease caused by mutations in the cystic fibrosis transmembrane conductance regulator (CFTR) gene, with pediatric onset and a median life expectancy of 36.8 years. Over 70,000 people are affected by CF in Europe and the United States, and around 2000 different mutations in CFTR gene have been described. Unfortunately, it is found in a wide range of phenotypes, and affected organs that are not always explained by the genotype–phenotype relation, leading experts to postulate whether CF could be the result of multiple combining effects such as modifier genes or epigenetics [21,83].

**Figure 2:**
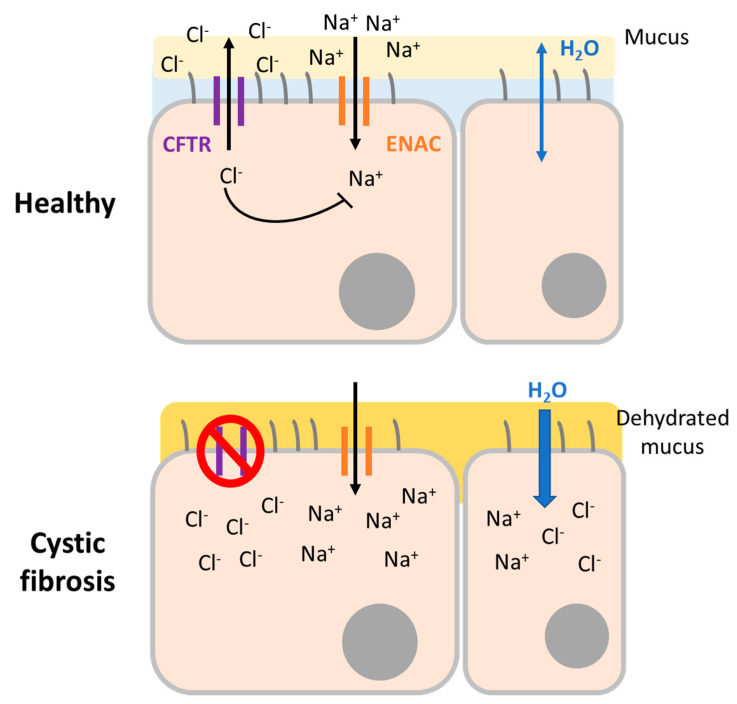
Pathophysiology of CF. Comparison between airway surface from a healthy (top) individual and a CF patient (bottom). In a normal situation, the cystic fibrosis transmembrane conductance regulator (CFTR) channel secretes chloride ions and inhibits sodium ions’ entrance, allowing water to hydrate the mucus by osmosis. When CFTR is mutated, ions are kept inside the cell and water moves inside the cell by osmosis, dehydrating the mucus and impeding mucus clearance from the airways.

The CFTR protein is a chloride and bicarbonate channel expressed in epithelial cells located in different organs. Dysfunctional CFTR provokes abnormalities in epithelial electrolyte transport leading to complications in the lungs, intestine, colon, liver, reproductive system, salivary, and sweat glands. In general, CF is characterized by increased salt concentration in sweat and thickened secretions in affected systems (Figure 2). However, specific symptoms can vary from chronic respiratory tract infections due to thick mucus immobility, chronic sinusitis, nasal polyps, bronchiectasis, and abnormal chest computed tomography to pancreatitis, failure to thrive or fat malabsorption, and many others. Among all these affected organs and symptoms, it is respiratory disease that provokes the most severe effects and is the main cause of CF-related death. Historically, CF diagnosis was based on sweat chloride levels and clinical symptoms. Today, it is confirmed by the presence of characteristic symptoms and evidence of dysfunctional CFTR. Over time, a tendency has emerged toward molecular diagnosis that can detect both symptomatic and pre-symptomatic patients [83].

Mutations described for CF have been organized into five groups. Class I mutations provoked the partial or complete absence of CFTR protein due to nonsense or frameshift mutations or mRNA splicing defects. Class II mutations are defects in protein processing causing the partial or complete absence of CFTR. The most common CF mutation, ΔF508, belongs to this group and results in complete absence of the protein. Class III mutations are called gating mutations because the resulting channels are resistant to normal activation and gating. Cass IV mutations affect transmembrane domains; the protein is functional but has a reduced capacity to conduct anions through the pore. Finally, class V mutations are found on the gene introns, so the protein is functional but the expression level is low or zero. This wide variety of mutations complicates finding a single drug to treat all CF patients. However, in 2012, the US Food and Drug Administration (FDA) approved ivacaftor, a CFTR potentiator, to treat patients with class III G551D mutation. In combination with lumacaftor (trade name Orkambi), this can treat ΔF508 homozygous patients. Although promising, these treatments are not indicated for all CF patients [84].

#### 3.1.1. Adenoviral Vectors

In the last few decades since the discovery of the CFTR gene in 1989 [85], many different strategies have been attempted to develop a gene therapy for CF. The first approaches arrived in the early 1990s, with adenovirus expressing CFTR cDNA. The partial restoration of chloride transport was detected, but patients reported nasal epithelium damage and immune response against the viral vector [86,87,88]. Recently, using a helper-dependent adenoviral (HD-Ad) vector expressing CFTR, in vivo mouse and pig airway basal cells and airway basal cells from CF patients in air–liquid interface (ALI) culture were transduced, and CFTR activity was restored [89].

#### 3.1.2. Lentiviral Vectors

Sendai virus (SeV) vectors efficiently transduce the apical surface of airway epithelial cells and achieve high gene expression levels. Both characteristics are very useful in CF gene therapy. Meanwhile, lentiviruses such as simian immunodeficiency virus (SIV) are able to transduce non-dividing cells, including airway epithelial cells. The SIV vector has been pseudotyped with key proteins of the Sev envelope: hemagglutinin-neuraminidase (HN) and fusion (F) protein. In addition, F/HN-pseudotyped SIV was optimized with the central popypurine tract (cPPT) and the Woodchuz hepatitis virus post-transcriptional regulatory element (WPRE) to drive CFTR gene expression. The respiratory mouse epithelium was transduced in vivo using a single formulation and without preconditioning, achieving beneficial expression levels. Long-term expression was detected, and readministration was also shown to be feasible. In addition, the F/HN-pseudotyped SIV vector was able to efficiently transduce human ALI cultures, where CFTR channel activity was restored [90]. Assessment of in vitro and in vivo models of toxicity, transduction efficiency, CFTR gene expression, protein functionality, genome integration sites, immune response, and optimization of the enhancer and promoter indicated that this vector could progress to human clinical trials to treat CF [91]. Other lentiviral vectors have been shown to be efficient in restoring CFTR function in animal models. For instance, three newborn pigs received aerosolized feline immunodeficiency virus vector pseudotyped with GP64 envelope encoding CFTR (FIV-CFTR). Two weeks after, functional CFTR, increased airway surface liquid pH and an increase also in bacterial killing was detected [92].

#### 3.1.3. Recombinant Adeno-Associated Viral Vectors

Outcomes of pre-clinical studies in animal models with recombinant adeno-associated virus serotype 2 encoding CFTR (AAV2-CFTR) resulted in a long sequence of clinical trials (Table 5).

The first one applied in humans consisted of AAV2-CFTR or placebo administration to the nasal epithelium of 12 CF patients. Even though gene transfer efficiency was very low, this study demonstrated that viral therapy was safe [92]. A phase I/II clinical trial concluded that AAV2-CFTR applied on the maxillary sinus was safe and has a dose-dependent effect on chloride secretion without immune response after 10 weeks [93]. Based on these results, a phase II trial was performed in patients who had undergone antrostomy. In one maxillary sinus, AAV2-CFTR was administered and in the other sinus, placebo was administered. Nevertheless, after 90 days, no significant differences were detected. Only anti-inflammatory interleukin-10 (IL-10) was increased in treated sinus. Gene therapy with AAV2 proved again to be safe but not efficient [94]. After a phase I trial to prove the safety of one-dose nebulization of the vector [7], a phase II trial assessed the safety of repeated aerosolized doses in CF patients, showing a decrease in interleukin-8 (IL-8) and an improvement of Forced Expiratory Volume in the first second (FEV1) measurement [95]. However, a phase IIb of this study with a higher number of participants did not prove any amelioration in CF patients, indicating that AAV2-CFTR did not improve lung function in the study conditions [96]. Other administration routes were tested, such as bronchoscopic procedures to reach the lower lobe of the lung, but this resulted in adverse effects related to bronchoscopy, minimal vector shedding, and PCR was only positive in the highest doses [8].

Due to the good safety profile but low expression level of transgene achieved by AAV2-CFTR, new adeno-associated virus serotypes have been investigated. A pseudotyped AAV5 was designed with chicken β-actin (CBA) promoter encoding a CFTR minigene (rAAV5-Δ264CFTR), given that the whole CFTR gene is bigger than the insert capacity of AAV5 vector. Either rAAV5-Δ264CFTR or rAAV5-GFP was aerosolized to macaques. Transfection efficiency, transgene expression, and CFTR protein detection were positive for both Δ264CFTR and GFP in all lung regions of the treated macaques in the absence of inflammatory response [97]. AAV1 has also shown to be more efficient in transducing cells and less immunogenic in chimpanzees and primary human airway cells.

AAV1 has also shown to be more efficient than AAV5 in transducing respiratory cells and less immunogenic when tested in chimpanzees. Both events are independent from one another, since AAV1 transfection efficiency is also higher than AAV5 in ALI culture [98]. Another study proved that AAV6 may be more efficient than AAV2 in mice for gene therapy targeting lung diseases [99]. In a different approach, Excoffon et al. [100] used PCR-based mutagenesis combined with a high-throughput in vitro recombination to create a library of chimeric capsid genes from AAV2 and AAV5. These variants were used to infect the human airway epithelial in ALI culture in order to select the most infectious form: AAV2.5T. This novel chimera vector encoding a shortened CFTR gene was able to restore chloride transport in CF airway epithelia [100]. Similarly, after in vivo selection of successful AAV2 evolved forms in pigs, the AAV2H22 high-efficient capsid was created. AAV2H22 expressing CFTR cDNA transduced to pig airways was able to restore chloride transport, but also to improve bacterial killing capacity [101].

Another possibility to tackle CFTR transduction is pre-mRNA segmental trans-splicing, where two halves of CFTR cDNA are encoded in two AAV6.2 vectors. After infection, the two pre-mRNAs form a full CFTR mRNA. CFTR function was restored in FRT-YFP cells (CFTR negative) and in IB3-1 cells, an airway epithelial cell line derived from an individual with CF [102].

#### 3.1.4. Non-Viral Vectors

Studies have also explored the option of non-viral therapies. For example, the plasmid called pGM169 expresses the CFTR gene under the control of human cytomegalovirus enhancer/elongation factor 1α sequence promoter. This plasmid was treated with cationic lipid GL67A to obtain pGM169/GL67A preparation (Table 5). A first preliminary clinical trial (NCT00789867) assessed the safety and efficacy of a single-dose nebulized administration of pGM169/GL67A [102]. Based on this study, a phase IIb trial performed repeated nebulization each 28 days for a year with 5 mL of pGM169/GL67A in CF patients (NCT01621867). At the end of the trial, a modest improvement in lung function, measured by Forced Expiratory Volume in the first second (FEV1) value, was observed in pGM169/GL67A group compared with the placebo group, and no serious adverse effects were reported [103].

A different non-viral approach was to combine a plasmid carrying CFTR gene with polyethylene glycol (PEG)-substituted 30-mer lysine polymers to form DNA nanoparticles that were administered to CF patient’s nostrils. After 14 days, there were no serious adverse effects. The measurement of nasal potential difference evidenced a partial completion of CFTR channel reconstitution in some patients, and PCR analysis demonstrated a mean of 0.58 vector copies/cell [104]. Although initial data are encouraging, more research is needed before development of a non-viral gene therapy that can be efficiently applied to CF patients.

#### 3.1.5. CFTR Gene Correction

Since viral and non-viral vectors failed to reach desirable efficacy outcomes as gene therapy, novel strategies for treating CF were developed as new editing tools appeared. ZFN have been applied to human bronchial epithelial (HBE) and CF tracheal epithelial (CFTE) cell lines, demonstrating that CFTR ΔF508 mutation, the most frequent among CF patients, was a suitable target and can be edited by both NHEJ and HDR [105]. ΔF508 and ΔI507 mutations were corrected with ZFN in iPSC from CF patients; after gene correction, the edited iPSC were derived to epithelial monolayer cells, where CFTR function was restored [106].

Two independent studies showed that ΔF508 mutation can also be corrected by iPSC electroporation from CF patients to introduce TALENs pairs and donor DNA. In one of them, iPSC were differentiated to airway epithelial submucosal gland cells that expressed a wild-type form of CFTR [107,108]. Given the many mutations that are described for CF, Xia et al. opted to use a HD-Ad vector to deliver TALENs in the IB3-1 cell line to introduce CFTR minigene cDNA on the AAVS1 locus. CFRT mRNA expression and protein function correction was detected on transfected cells [109].

Finally, promising results can also be found relative to CRISPR/Cas9 and CF gene therapy. CFTR ΔF508 mutation was corrected by the CRISPR/Cas9 system in intestinal stem cell organoids from CF patients transfected with lipofectamine. Clonally expanded organoids showed corrected allele of the target gene and a functional protein [110]. The same mutation was also corrected in iPSC cells. A 20% correction rate was achieved using electroporation for transfection and introducing the CRISPR/Cas9 system as a ribonucleoprotein complex [111]. Following a different approach, three mutations found in 1.5% of CF patients that provoke alternative strong splice sites were assessed. Two gRNA targeting either side of each mutation were designed to produce two DSBs that resulted in the mutation excision in the region; thus, normal splicing was restored via NHEJ [112].

#### 3.1.6. Conclusion

In summary, CF is a monogenic but complex disease for which definitive treatment has not yet been established. As a consequence, since the CFTR gene was discovered as the cause of CF, there has been considerable interest in developing gene therapy, as exemplified by viral and non-viral vectors, aerosolized and intratracheal administration, single-dose and multiple-dose, wild-type gene integration, and gene editing tools. Although there are promising and hopeful breakthroughs in CF gene therapy, drawbacks such as the renewal of epithelial cells, difficult-to-transduce airway cells, and thick mucus in CF patients together with concerns about immune activity against delivery vectors must be resolved before gene therapy can be brought to clinical practice.

### 3.2. Alpha-1 Antitrypsin Deficiency, the Genetic COPD

Alpha-1 antitrypsin deficiency (AATD) is a rare genetic condition caused by mutations on the SERPINA1 gene, which is responsible for expression of the protein alpha-1 antitrypsin (AAT). Over 100 mutations have been reported to provoke AATD, but the most frequent defective alleles are called PiZ and PiS (the S mutant form of human AAT), while the wild-type form is called M.

**Figure 3:**
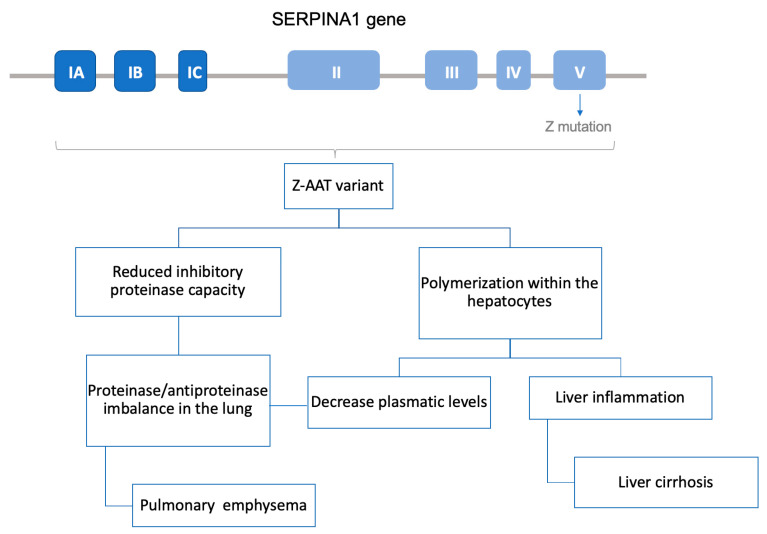
Schematic view of AATD associated with the Z mutant form of human AAT (PiZ) allele pathophysiology. At the top, the SERPINA1 gene scheme with the seven introns and the PiZ mutation in the V exon, where the active site is found. Z-AAT variant, codified by the PiZ allele, polymerized in the hepatocytes leading to a proinflammatory state in the liver that can produce cirrhosis and decrease AAT plasma secretion levels. In addition, the proteinase inhibitory capacity of Z-AAT variant is reduced, facilitating pulmonary emphysema development.

AAT is a glycoprotein whose main function is the inhibition of serine proteases such as neutrophil elastase. Around 80% of AAT is produced and secreted by the hepatocytes. Through systemic circulation, AAT diffuses to the lung, where it protects the tissue from the proteolytic activity of neutrophil elastase. In AATD patients with the PiZZ phenotype, mutated protein forms polymers inside the hepatocyte, which not only leads to a decrease in AAT circulating levels but also produces a chronic liver inflammation state that may result in cirrhosis. In the case of PiS mutation, the misfolded protein is degraded before being secreted and does not reach the bloodstream. Therefore, both PiZ and PiS precipitate a decrease in AAT concentration in the pulmonary tissue. As a result, the lungs are left unprotected from neutrophil elastase, creating a protease–antiprotease activity imbalance and thus facilitating the destruction of parenchymal lung. Consequently, patients often develop chronic obstructive pulmonary disease (COPD); in fact, AATD is known as the genetic COPD, because it is the main genetic cause of this highly common disease (Figure 3).

The only approved treatment for AATD is called augmentation therapy. Based on intravenous infusions of purified AAT, it is only able to slow the progression of emphysema and is expensive and time-consuming for the patient [59].

AATD is a complex disorder presenting a wide range of symptoms and severity. The most severe condition is associated with the PiZ allele. Ninety-six percent of diagnosed AATD cases have the PiZZ phenotype, and the remaining 4% are mostly PiSZ [113]. These data can be explained by the fact that it is a highly underdiagnosed disorder, above all in patients with mild symptoms, similar to other prevalent diseases, that usually have PiMS or PiSS phenotypes.

The fact that it is a monogenic disease and that PiZ, the most frequent allele, is a well-characterized point mutation produced by a G > A substitution at codon 342 (Glu342Lys) in exon 5 of SERPINA1 gene make AATD a suitable target for gene therapy [114]. Overall, two different approaches have been performed: gene augmentation therapy by introducing the M allele sequence in the organism and correction of the PiZ allele by gene editing.

#### 3.2.1. Viral Gene Augmentation Therapy

The first gene augmentation therapy attempts used retroviral vectors to deliver AAT into cell lines. Results were promising, but when performed in vivo in animal models, retroviral therapy produced severe adverse effects due to insertional mutagenesis. To avoid these problems, research focus then turned to adenoviral vectors, which do not integrate in the genome. Some in vivo applications in animal models were reported, but they showed two major drawbacks: high immunogenicity and transient protein expression [9].

In the late 1990s, adeno-associated viral (AAV) vectors began to be used in AATD gene therapy. AAV vectors were first tested in different mouse strains with two different promoters via intramuscular injection. It was found that in vivo, cytomegalovirus (CMV) promoter achieved therapeutic and long-term serum levels of human AAT in C57BL6 mice, which were even higher in severe combined immunodeficiency (SCID) mice [115]. These promising data lead to a phase I clinical trial where an rAAV serotype 2 expressing AAT (rAAV2-AAT) vector was administered via intramuscular injection in 12 adults with AATD with at least one PiZ allele. Only low levels of wild-type AAT were achieved, but high safety levels were proven, and although antibodies against rAAV2 were detected, there were no associated adverse effects [116]. In order to improve the efficiency of transgene expression but maintain the safety profile, another phase I clinical trial was launched switching to a rAAV1-AAT vector. One year after administration, M-AAT levels were sustained in two subjects, although 200-fold below the therapeutic level. Although T cell response against rAAV1 capsid was developed within the first 14 days after treatment, no elevation of creatine kinase was detected, so it was concluded that immune response did not completely eliminate transduced myofibers, which allowed the sustained expression of M-AAT over time [117]. In a phase II clinical trial, the same vector was produced by a herpes simplex virus complementation system and administered by intramuscular injection to nine adults with AATD. As in the previous trial, an immune response to AAV capsids was detected, but not against M-AAT. M-AAT levels were dose dependent, peaked at day 30, and then decreased to 3–5% of target concentration for at least 90 days [118]. Five years after the single administration, similar results were obtained. Patients continue to show a persistent but low expression of M form of AAT and a regulatory T cell response that did not provoke adverse effects. Beneficial effects on neutrophil activity such as elastase inhibition and degranulation were also reported [119].

Aiming to circumvent human immune response against rAAV2 and to optimize cell transduction and AAT expression efficiency, alternative administration methods and new AAV serotypes have been explored. The intrapleural administration of rAAV2-hAAT and rAAV5-hAAT in C57BL/6 mice showed that this method generated higher lung and serum levels of hAAT than intramuscular administration and that rAAV5-hAAT was 10-fold more efficient than rAAV2-hAAT in both pleural and intramuscular administration [120]. As a consequence, another study screened 25 AAV vectors derived from human and non-human primates [121]. After intrapleural administration in mice, analysis of AAT serum levels showed that AAV rhesus macaque-derived serotype 10 (AAVrh.10-AAT) was the most efficient vector, and as humans are not exposed to it, there are presumably no problems of previous sensibilization. These results led to an ongoing phase I/II clinical trial called ADVANCE, which assessed two different doses of AAVrh.10-AAT intrapleural administration in individuals with AATD to evaluate the safety and changes in AAT expression in serum and liver (ClinicalTrials.gov Identifier: NCT02168686) [9].

Other approaches for gene augmentation therapy have been applied to in vitro and in vivo mouse models reporting potential strategies to increase serum protein concentration. Stoll et al. [122] created a fusion plasmid vector that contains the Epstein–Barr virus nuclear antigen 1 (EBNA1) gene sequences and the full length *SERPINA1* genomic sequence encoding hAAT. This plasmid showed high expression efficiency when transfected in vitro human and mouse cell lines and also when applied in vivo by hydrodynamic tail-vein injection in mice. Invasive methods such as intratracheal administration of lentivirus or rAAV8 expressing the M allele of hAAT have shown to produce high levels of hAAT in mice lung cells and to ameliorate pulmonary emphysema [123,124]. Recently, in vivo intravenous injection in mice tails with an adenoviral delivery of CRISPR/Cas9 achieved hAAT integration into the ROSA26 safe harbor. Gene integration led to the long-term detection of hAAT in mice serum and liver cells [125]. The autologous transplantation of ex vivo transduced cells with lentiviral or AAV vectors encoding hAAT resulted in sustained hAAT circulating levels in mice [126,127]. Hepatocyte-like cells derived from human mesenchymal stem cells were successfully transduced with a lentiviral vector encoding AAT as a potential source of cells for possible future autologous transplant in the clinical management of AATD [128].

#### 3.2.2. Dual Therapy Approach: Addressing Hepatic and Respiratory Disease with MiRNA

Up to now, our review has focused on various attempts to increase AAT circulating levels; however, AATD is also characterized by the harmful polymerization of Z-AAT forms in hepatocytes, and a complete cure for this syndrome should therefore tackle both hepatic and respiratory symptoms. To assess this dual therapy strategy, Mueller et al. have developed several rAAV vectors containing microRNA (miRNA) targeting the mutant AAT gene and also expressing a miRNA-resistant M-AAT allele. Transgenic mice expressing human Z-AAT were treated with a dual-function rAAV9 vector. Z-AAT aggregates were reduced in hepatocytes at the same time that Z-AAT levels in serum decreased by 80% and M-AAT levels were increased. In addition, the miRNA profile was not altered, suggesting that this miRNA-based dual approach could be safe and efficient to treat AATD, in contrast to small interfering RNA (siRNA) and short hairpin RNA (shRNA) based-therapies that have previously reported side effects [129].

#### 3.2.3. Non-Viral Therapy

**Figure 4:**
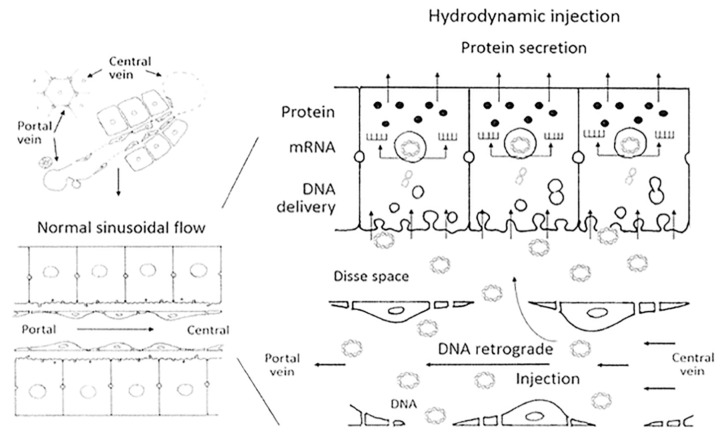
Schema of hydrodynamic transfection within liver tissue in pigs. Retrograde injection of DNA through the infrahepatic inferior vena cava produces a separation between endothelial cells and their fenestration and the formation of endocytic vesicles, allowing DNA molecules to enter the hepatic cells. Modified with permission from Sendra et al. [131] under the terms of the Creative Commons Attribution License (https://creativecommons.org/licenses/by/4.0/).

Safety concerns about viral vectors have encouraged many researchers to develop other non-viral gene delivery methods for gene therapy. One example is the hydrodynamic procedure, which is based on the rapid intravenous injection of a large volume of a naked DNA solution to transfect mainly liver cells in small animals [28]. Long-term therapeutic levels of hAAT were achieved in mice via the hydrodynamic procedure by transfecting liver cells through the tail vein with the pTG7101 plasmid. This plasmid encodes the full length of the hAAT gene under the control of its natural promoter. The AAT plasma concentration reached therapeutic levels and remained stable for at least 20 days. Furthermore, liver cells showed hAAT expression four months after treatment [130]. However, hydrodynamic transfection has some hemodynamic adverse effects that must be circumvented for this method to be considered in clinical practice. To do that, larger animal models such as pigs have been used to set up a new hydrodynamic approach whereby the liver was surgically sealed and pTG7101 delivery was subsequently performed through the infrahepatic inferior vena cava directly to the liver. The hepatic expression of hAAT was achieved, but at lower levels than in mice, and AAT plasma presence could unfortunately not be detected, which is possibly due to a species-related issue (Figure 4) [131]

Hydrodynamic gene delivery is a promising tool for liver genetic modification to treat AATD and other hepatic pathologies, especially since ex vivo human liver segments have been efficiently transfected with the IL-10 gene [132].

### 3.3. SERPINA1 Gene Correction

The gene correction of *SERPINA1* mutations in hepatocytes is another option that could provide a definitive therapy to cure AATD. It still needs more in-depth research, but some steps toward gene correction therapy have already been taken. Yusa et al. [133] were able to correct PiZ mutation in both alleles of iPSC from patients with AATD. iPSCs were cotransfected with ZFN targeting PiZ mutation and a puromycin resistance cassette flanked by piggyBac repeats, which after the selection of modified colonies is removed using piggyBac transposase transient expression. This method proved capable of correcting both alleles in 4% of colonies and had little off-target activity. Edited iPSCs were transformed in vitro to hepatocyte-like cells. These hepatocyte-like cells maintain their biological functions, did not form polymers of mutant AAT, and secreted functional AAT. They were successfully transplanted via intrasplenic injection to mice liver, where they integrated into the mouse liver parenchyma, did not form tumors, and expressed M form of hAAT.

Some years later, a similar study was published, also transfecting iPSC with the same integrating vector, but in this case with TALEN constructs, in which the results obtained were successful. All selected clones after gene correction and the excision of selection cassette showed that both PiZ alleles changed to the M sequence. When differentiating iPSC to hepatocyte-like cells, they showed no alteration of growth pattern nor metabolic capabilities. The aggregation of mutant AAT was not detected; instead, levels of wild-type AAT as measured by ELISA assay were normal [114].

CRISPR/Cas9 have also been applied to iPSC lines to correct PiZ mutation of the SERPINA1 gene. In the study discussed below, Smith et al. investigated the relative efficiency of CRISPR/Cas9 and TALENs for inducing NHEJ and HDR. Most relevant for this review is the fact that PiZ point mutation was chosen to perform the experiments. iPSC cells were transfected by electroporation with 4D-Nucleofector equipment (Lonza) with the designed TALENs constructs or with two plasmids: one expressing Cas9 protein and the other expressing the sgRNA. The percentage of indel produced was around 2% for CRISPR/Cas9 but was undetectable for TALENs. Curiously, HDR efficiency was very similar across both systems. TALENs showed a relative preference for HDR over NHEJ, while CRISPR/Cas9 was prone to NHEJ. In addition, sgRNA proved to be allele-specific. In heterozygous PiMZ iPSC lines, the CRISPR/Cas9 system was able to discriminate and specifically target PiZ allele [134].

With regard to animal models, the CRISPR/Cas9 system has been delivered with an adenoviral vector to a mouse model of human AATD which expresses the PiZ allele of hAAT. In this study, PiZ expression was disrupted via the NHEJ reparation pathway after the DSB produced by Cas9. Circulating hAAT levels were reduced by 94% at nine weeks after treatment. Liver biopsies showed a significant decrease in AAT aggregates and a decrease in liver fibrosis and inflammation. Obviously, this study does not address the reversal of AATD pathology, but it nonetheless opens a door to research in CRISPR/Cas9 gene editing in vivo treatment to reduce mutant protein, although potential risks must be carefully investigated [135].

#### Conclusions

In conclusion, gene therapy is a promising substitute to the existing and controversial augmentation therapy, as evidenced by the encouraging results in both pre-clinical and clinical phases. Research is centered mainly on AAV vectors. Despite the safety of these vectors, AAT expression remained far below the minimum therapeutic level. Some projects in animal models are working on optimizing this therapy. Furthermore, new gene editing tools used to correct mutations are trying to find a definitive cure for AATD, but for the moment, testing remains at the animal and in vitro model stage, as there still are many concerns about safety and off-target activity.

## 4. Primary Ciliary Dyskinesia

**Figure 5:**
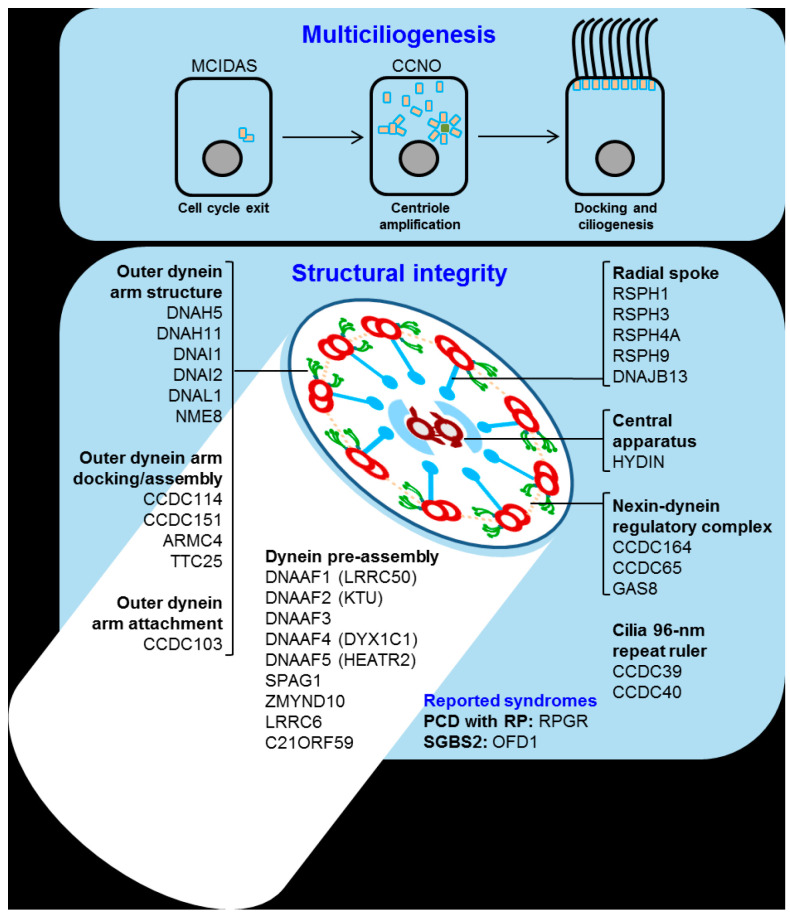
Structure of motile cilia and role of mutant proteins. Motile ciliopathies are caused by mutations in the (top panel) components of the ciliogenesis pathway or (bottom panel) structural and attachment proteins of the axoneme dynein ‘arm’ motors (green), the dynein arm docking complex, the nexin–dynein regulatory complex (dotted lines), the central apparatus (brown), the radial spokes (blue), as well as molecular ruler proteins and cytoplasmic dynein arm assembly factors. Reported syndromes are: primary ciliary dyskinesia (PCD) associated with retinitis pigmentosa (RP), and Simpson–Golabi–Behmel syndrome, type 2 (SGBS2). Reproduced with permission from [136].

Primary ciliary dyskinesia (PCD) is a rare genetically heterogeneous disease that causes the disrupted movement of motile cilia in the organism. This disruption is generally related to poor mucociliary clearance and long-term lung function distress. The main cause of the PCD is inherent to the cilia. Over 600 proteins form its ultrastructure, and the malfunction of any of the genes related to forming or docking the structure could impair movement (Figure 5).

Thus, over 40 genes are known to be related to PCD. Of the total diagnosed patients, around 70% have a mutation in one of those 40 genes, while in the remaining 30%, the origin is unknown [137]. The most common mutations found in patients are located in the outer dynein arm (ODA) docking proteins, with DNAH5 mutations being the cause of around 25% to 30% of all PCD cases [138,139,140]. Other ODA-related genes are also commonly mutated in PCD such as DNAI1 (approximately 10%), DNAH11 (approximately 6%) and DNAI2, which are genes related to radial spokes (RSPH4A, RSPH9) and other genes involved in ciliary assembly (KTU, LRRC50) [141].

The complexity and heterogeneity of this disease is a major concern when testing new approaches to find a suitable gene therapy. Due to the multigenic nature of the disease, finding a single drug that can treat the whole patient pool is highly unlikely, so personalized medicine will play a key role in PCD.

Restoring cilia motility in PCD using genetic tools started back in 2009 with studies in the ODA gene DNAI1. DNAI1 is a PCD-related gene whose deficiency has been observed to cause a loss of ODA structures and thus impair cilia motility. Incidence is estimated to be 10% of PCD cases. Dnaic1 is the murine DNAI1 orthologue, so Dnaic1-deficient cells show a lack of ODA in ALI culture. In order to mimic PCD, Dnaic1^−/−^ mice were created using tamoxifen-inducible Cre-Lox technology to overcome embryo development complications. When tamoxifen is applied, Dnaic1^−/−^ mice show reduced mucociliary clearance and chronic rhinosinusitis [142].

The transduction of Dnaic1^−/−^ undifferentiated ALI culture with a full-length Dnaic1 cDNA encoded in a lentiviral vector showed a visible increase in ciliary activity, from approximately 0.4% to 10%, and the CBF remained roughly the same. On the other hand, the transduction of a differentiated ALI culture also showed a surface area increase from approximately 1.0% to a maximum of 8.8% and a 10-fold increase in ciliary activity. On differentiated cultures, active ciliated cell appearance showed a 5–6 day delay after treatment.

The tamoxifen-induced deletion of Dnaic1 in a murine model showed that less than 20% of normal gene expression could restore a significant mucociliary clearance and reduce the severity of the disease. To observe gene transfer in the airway epithelium, a luciferase and β-gal lentiviral vector was administered by nasal inhalation. Vector infection rates in PCD mice showed an 8-fold drop compared to control. This inhibited gene transfer was afterwards attributed to the chronic rhinosinusitis [19].

In 2016, new gene therapy approaches using TALENs were used for the ex vivo restoration of the DNAH11 gene. Ciliated cells were collected via nasal brushing from PCD patients heterozygous for a known mutation (p.R2250*/p.Q3604*). Left and right TALENs were designed to target p.R2250*, and after successful design, they were cloned into LAW34 lentivirus, which is a feline immunodeficiency virus (FIV) vector.

**Figure 6:**
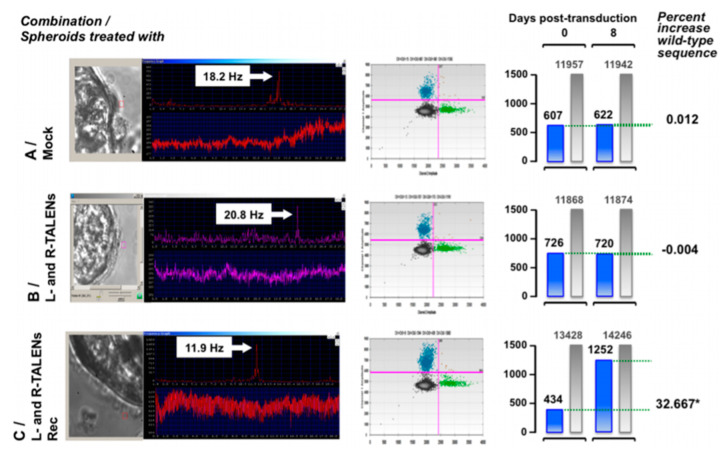
Analysis of ciliary motility and number of wild-type alleles at day 0 and 8 post-transduction. Spheroids from patient A were transduced with the vectors indicated on the left and monitored every 2 days for ciliary beating pattern and frequency. Panels show the ciliary beat frequency measured at day 5 post-transduction for untreated cells (upper panel), cells treated with L-and R-transcription activator-like effector nucleases (TALENs) (middle panel), and L-and R-TALENs and Rec (lower panel). The latter treatment reduced beating frequency to normal levels (12–14 Hz). Normalization lasted throughout the observation treatment. At day 0 and 8 post-treatment, spheroids were collected in part and processed to extract the genomic DNA. Extracted nucleic acids were analyzed by droplet digital PCR (ddPCR) to count the number of molecules containing the wild-type sequence at nucleotide position 172 381. The center gray panels show, spatially separated, the wild-type sequence molecules in the top left quadrant (blue dots), mutated sequence molecules in the lower right quadrant (green dots), and irrelevant molecules in the lower left quadrant (gray dots) counted by ddPCR. The histograms on the right show the number of wild-type sequence molecules counted in spheroids at day 0 and 8 post-transduction and the percentage increment increase in wild-type molecules between the time points. Spheroids treated with L- and R-TALENs and Rec show >30% increase in wild-type molecules. This increase reached statistical significance (*) as determined by the sequential probability ratio test (α value 0.01). Reproduced with permission from [143].

Nasal brushings from the patients were cultivated in spheroids containing the ciliated cells inside. Six to eight days after obtaining the spheroids, the lentiviral vector containing the TALEN sequences was added to the media and successfully corrected the mutated sequence to the wild-type one, as proven by molecular analysis, evaluation of ciliary beat phenotype, and visual examination of cells treated for immunohistochemistry (Figure 6) [143].

In conclusion, there is still a long road ahead to find a suitable treatment for PCD, but by deepening our insight into cilia biology, its role in molecule trafficking and its assembly, we have created new approaches for restoring motility.

## 5. Discussion

Nowadays, there are numerous tools available to conduct a gene therapy experiment. Endogenous genes can be modified, edited, disrupted, inhibited, and enhanced; likewise, exogenous genetic material can be introduced into the cell as an episome or integrated in the genome. Many different organs can be targeted via different administration routes; theoretically, cells can even be modified ex vivo and then implanted back in the same individual. Extensive research effort has gone into bringing gene therapy from bench to bedside and hundreds of clinical trials have been performed; however, only a few gene or cell therapies are approved at the moment. Yet during the last few decades, especially since the development of CRISPR/Cas9 technology, there is renewed hope in gene therapy as a treatment for genetic conditions.

As regards respiratory diseases, many of them are chronic and caused by genetic defects. The lung is an organ accessible to gene therapy, but it also presents physical and immunological barriers that impair gene delivery, especially for viral transduction. Aside from physiological barriers, symptomatology such as a thick mucus layer in CF or rhinosinusitis in PCD complicate the process. In addition, to achieve the long-term expression needed in chronic conditions, repeated administrations are needed because of the constant cell renewal in the lung, thus potentially stimulating the immune response. As observed in CF and AATD clinical trials with rAAV vectors, efficacy in gene delivery is very low, and although there are no related adverse effects, there is an immune response to the viral capsid. To circumvent this problem and obtain permanent effects with a unique administration therapy, respiratory stem cells should be targeted, requiring integrative vectors that can produced insertional mutagenesis. Furthermore, gene delivery into these cells in vivo is an important limitation, as they are located in the basal surface of distal airways, and available vectors are not able to reach them efficiently. Undoubtedly, lung stem cell gene delivery is one of the main future challenges for gene therapy development.

In the case of CF and AATD, rAAV vectors have turned out to be the most efficient and safe for therapy, and many clinical trials have been run using them as drugs. However, research is focused on optimizing transfection efficiency by pseudotyping and optimizing promoters and enhancers, such as F/HN-pseudotyped SIV, or looking for new serotypes, such as the AAVrh.10. Conversely, the most frequently used vector for PCD is the lentiviral type. Target cells for PCD therapy are ciliated epithelial cells, which are non-dividing cells that only can be infected with lentivirus or retrovirus.

Alternatively, non-viral vectors can also be used for gene therapy. They are safer but also less efficient than viral vectors. A handful of non-viral vector clinical trials can be found using plasmids encoding CFTR gene combined with cationic lipids and PEG, with modest results. The hydrodynamic procedure has shown encouraging outcomes to treat AATD in pre-clinical studies in vivo, but there are many concerns about the hemodynamic effects if applied to humans. In contrast, non-viral vectors are very useful for in vitro or ex vivo applications.

Gene augmentation therapy is the most widely used approach to treat CF, AATD, and PCD. However, the development of gene editing tools has opened up a new range of possibilities in gene therapy, one of the most promising of which involves editing iPSC ex vivo and then inducing differentiation in the target cell of interest, and finally, autotransplantation with corrected cells. Recently, a number of clinical trials using CRISPR/Cas9 have been approved for oncologic conditions. Pending the results of these trials, the successful application of gene editing tools in CF, AATD, and PCD might be the first step toward definitive and personalized treatment.

In conclusion, the three conditions considered in this review are rare genetic respiratory diseases with complex clinical expression and without definitive treatment. For all three, gene therapy is an encouraging alternative treatment to conventional drugs that have been inefficient up to now. As discussed in this review, recent decades have seen great strides in applying gene therapy in these respiratory conditions; however, researchers are still working toward new breakthroughs due to ongoing concerns about safety, specificity, and efficacy.

## Figures and Tables

**Table 1:** Viral vectors used in gene therapy reporting genome integration capability and summary of main advantages and drawbacks. The “studies in CF, AATD, PCD” column shows if there are any in vitro or in vivo studies using the viral method. * Herpes simplex virus is used as a complementation method for the adeno-associated virus. CF (Cystic fibrosis), AATD (Alpha 1 antitrypsin deficiency), PCD (Primary ciliary dyskinesia).

Viral Vector	Genome Integration	Advantages	Disadvantages	Studies in CF, AATD, PCD
*Lentivirus*	Yes	Long-term expression	Mutagenesis potential	Yes [17], yes [18], yes [19]
*Retrovirus*	Yes	Long-term expression in dividing cells	Mutagenesis potential	Yes [20], yes [18], no
*Adenovirus*	No	Transduction is efficient in many cells	Strong antiviral immune response	Yes [21], yes [22], no
*Adeno-associated virus*	No	Non-inflammatory and non-pathogenic	Requires helper virus Small packaging capacity	Yes [21], yes [18], no
*Herpes simplex virus*	No	Large capacity	No expression when the infection is latent Tropism to neurons	No, yes [23] *, no

**Table 2:** Physical methods used for DNA insertion into cells with a summary of the main advantages, disadvantages, and the physical principle underlying the method.

Physical Methods	Advantages	Disadvantages	Principle
Microinjection	Specific delivery Safe, Simple	Low efficiency	Uses a needle to inject the material [27]
Ballistic DNA	Precise delivery	Limited applications	Application of a pressurized gas to introduce nanoparticles in the cell [28]
Electroporation	Good efficiency Reproducible results	Low viability	Uses a high-voltage electric current to destabilize membrane polarity [29]
Sonoporation	Safe, Flexible	Cell damage	Ultrasounds create pores in the membrane [28]
Photoporation	Theoretically very efficient	Expensive Complex	Use of highly concentrated light beams that perforate the membrane [28]
Magnetofection	Specific delivery Used for difficult to transfect cells	Expensive Complex	Uses magnetic fields to move magnetic-sensitive particles to the cells of interest [30]
Hydroporation	Safe, Simple	Low efficiency Complex in large animals	Exerting osmotic pressure in the tissue environment Promotes particle movement to the interior of the cells [28]
Mechanical Massage	Safe, Simple	Low efficiency	Mechanical movement of the liver makes it permeable to DNA and nanoparticles [31]

**Table 3:** Inorganic particles used to stimulate DNA insertion into the cells with a summary of the main advantages, disadvantages, and the chemical principle underlying the method.

Inorganic Particles	Advantages	Disadvantages	Principle
Calcium Phosphate	Biocompatible Biodegradable	Might crystalize when stored	Calcium is naturally absorbed by the cell [33]
Silica	Low toxicity Easy to store Very versatile	Interacts with serum proteins	Silica-functionalized nanoparticles are recognized and engulfed by cells [34]
Gold	Inert High transfection efficiency	Accumulation Long-term effects have not been studied	Its small size allows it to permeate the cell, the near infrared light absorption can be used for selective delivery [35]

**Table 4:** Biocomponents used to stimulate DNA insertion into the cells with a summary of the main advantages, disadvantages, and the chemical principle relying on the method.

Biocomponents	Advantages	Disadvantages	Principle
Cationic lipids	Flexible	Can become toxic at certain concentrations	Positive charges interact with the negative charged proteoglycans and glycoproteins in the membrane, helping the particles of interest enter the interior of the cells [36]
Lipid Nano Emulsions	Stable Very low toxicity	Less toxic than cationic lipids	
Solid lipid particles	Increased protection for the delivery material	Complex to produce	
Peptide based	Multifunctional Specific Safe	Complex to produce	Peptides can be added to lipoparticles for specific recognition or delivery [37]
Polyethylenimine	Widely used	Difficult to use in in vivo models	Increases osmotic pressure in the cell, creating pores in the membrane [38]
Chitosan	Non-toxic Mucoadhesive	Low efficiency	Increases osmotic pressure in the cell [39]
PLA/PLGA	Small, Phagocyted	Can induce immune reaction	Biodegradable polyesters that deliver their content by hydrolysis [40]
Dendrimers	Flexible, Good interaction	Toxicity	Small size allows them to interact with cell membranes, favoring DNA uptake in cells [41]
Polymethacrylate	Small	Poor membrane interaction	Small size allows them to reach the whole organism and deliver the content [42]

**Table 5:** Summary of clinical trials performed in CF with tgAAVCG viral vector and pGM169/GL67A non-viral vector. FEV1: Forced Expiratory Volume in the first second, IL: interleukin.

Clinical Trial	Vector	Administration Route	Outcome	References
Phase I	rAAV2-CFTR (tgAAVCG)	Nasal epithelium	Low gene transfer efficiency Safety profile	Flotte et al. (1996)
Phase I/II		Maxillary sinus	Dose-dependent effect Safety profile	Wagner et al. (1998)
Phase II		Maxillary sinus	No significant differences Safety profile	Wagner et al. (2002)
Phase I		One-dose nebulization	Safety profile	Aitken et al. (2001)
Phase II		Repeated-dose nebulization	Decrease of IL-8 Improvement of FEV1	Moss et al. (2004)
Phase IIb NCT00073463		Repeated-dose nebulization	No improvement of lung function	Moss et al. (2007)
Phase I NCT00004533			Adverse effects Minimal vector shedding PCR positive only in highest dose	Flotte et al. (2003)
Phase I/IIa NCT00789867	pGM169/GL67A	One-dose nebulization	Safe and efficient	Alton et al. (2015)
Phase IIb NCT01621867		Repeated-dose nebulization each 28 days for one year	Modest improvement in FEV1 value No adverse effects	Alton et al. (2015)
